# Dynamic imaging of interfacial electrochemistry on single Ag nanowires by azimuth-modulated plasmonic scattering interferometry

**DOI:** 10.1038/s41467-023-39866-8

**Published:** 2023-07-13

**Authors:** Gang Wu, Chen Qian, Wen-Li Lv, Xiaona Zhao, Xian-Wei Liu

**Affiliations:** 1 Chinese Academy of Sciences Key Laboratory of Urban Pollutant Conversion, Department of Environmental Science and Engineering, University of Science and Technology of China, Hefei, 230026 China; 2 Department of Applied Chemistry, University of Science and Technology of China, Hefei, 230026 China

**Keywords:** Catalysis, Imaging studies, Imaging and sensing

## Abstract

Direct visualization of surface chemical dynamics in solution is essential for understanding the mechanisms involved in nanocatalysis and electrochemistry; however, it is challenging to achieve high spatial and temporal resolution. Here, we present an azimuth-modulated plasmonic imaging technique capable of imaging dynamic interfacial changes. The method avoids strong interference from reflected light and consequently eliminates the parabolic-like interferometric patterns in the images, allowing for a 67-fold increase in the spatial resolution of plasmonic imaging. We demonstrate that this optical imaging approach enables comprehensive analyses of surface chemical dynamics and identification of previously unknown surface reaction heterogeneity by investigating electrochemical redox reactions over single silver nanowires as an example. This work provides a general strategy for high-resolution plasmonic imaging of surface electrochemical dynamics and other interfacial chemical reactions, complementing existing surface characterization methods.

## Abstract (web summary)

Direct visualization of the chemical dynamics of surfaces in solution is essential to gain mechanistic insights into nanocatalysis and electrochemistry. Here, the authors demonstrate the imaging of dynamic interfacial changes on a single nanowire during chemical reactions using azimuthally modulated plasmonic scattering interferometry.

## Introduction

Nanoscopic heterogeneities critically influence surface chemical processes on nanoparticles in electrochemical, environmental, and catalytic reactions^1–3^. For example, heterogeneous catalytic reactions often occur at the surfaces of solid catalysts and are dictated by their surface properties^4–6^. Spatially resolved probing of the dynamic changes in the active sites of a single nanoscale entity during chemical reactions, particularly in an aqueous solution, is crucial for understanding the structure-performance relationship and designing, developing and implementing new catalysts. Conventional spectroscopic techniques for in situ studies of chemical reactions can provide insight into the chemical processes but show limited spatial resolution at the micrometer scale due to the diffraction limit of light^7,8^. Atomic force microscopy (AFM) is useful for dissolution or crystallization imaging without the diffraction limit^9^. However, it is incapable of wide-field and high-throughput imaging of surface reactions over single entities in real time. Advanced synchrotron-based measurements can detect the chemical and structural changes of a single nanoparticle. However, such techniques are costly and time-intensive and can result in beam-induced sample degradation^2,4^. Single-molecule fluorescence spectroscopy has been used to image single turnover sites on catalytic surfaces but is restricted to reactions that generate highly fluorescent product molecules^10,11^. Light-activated electrochemistry^12,13^ and scanning electrochemical microscopy^14^ can image the local electrochemical current. However, they are not suitable for high-throughput and real-time visualization of surface chemical dynamics over single entities with a sub-particle spatial resolution due to the limited temporal resolution. It is therefore still a challenge to visualize dynamic interfacial changes over a single particle with high spatiotemporal resolution for mild solution reaction conditions.

Here, we report the method of azimuth-modulated plasmonic imaging that provides in situ images of dynamic electrochemical reactions over single nanowires in solution. This optical imaging method does not require extrinsic labels and allows for visualizing dynamic changes at active sites in real time. This is particularly useful, especially for monitoring reactions in solution, which is challenging for other state-of-the-art methods. To visualize the surface dynamics in situ over a single nanowire in real time, we imaged the interferometric pattern of a nanowire scattering the planar plasmonic wave propagating on its surface. The plasmonic-based imaging approach measures the surface reactions on a gold film rapidly^15–17^. Although there has been intense interest in mapping the heterogeneous distribution of surface sites of nanomaterials, real-time imaging of electrochemical reactions at the nanoscale is currently impossible with plasmonic imaging due to the micrometer spatial resolution and a large background signal arising from interference between scattering and reflected light^18^. We therefore propose a strategy for eliminating reflected light by modulating the incident light, which enabled plasmonic imaging with improved spatial resolution and anti-interference capability. We previously illustrated a proof-of-concept approach for identifying nanoparticles or detecting the compositional evolution of nanoparticles by developing a computational tool^19,20^. By integrating this tool with our strategy to modulate incident light, we present a methodology for high spatiotemporal resolution plasmonic imaging.

## Results

### Azimuth-modulated plasmonic scattering interferometric imaging

**Fig. 1:**
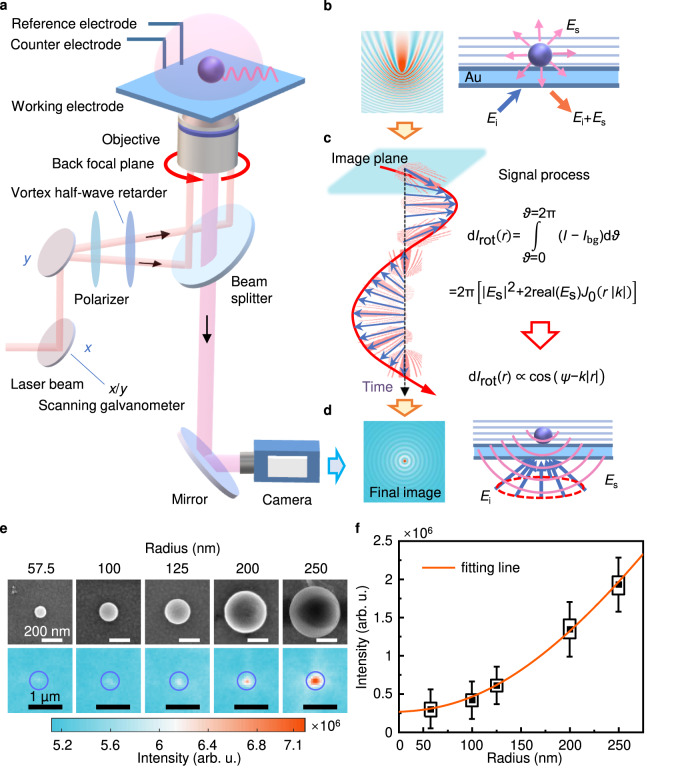
Principle of azimuth-modulated plasmonic scattering interferometric microscopy. **a** Schematic of the plasmonic imaging setup. The azimuth of the surface plasmonic wave was modulated by rotating the incident location of the beam on the back focal plane (red arrow) using a *x*/*y* scanning galvanometer. Scattering light was recorded with a CCD camera. **b** Principle of plasmonic scattering interferometric imaging. The objective-scattered surface plasmons (*E*_s_, pink arrows) interfered with the excited incident surface plasmon E-field (*E*_i_, light blue lines), resulting in a parabolic-like interference pattern. **c** Signal processing of plasmonic images. The integral of the modulated surface plasmonic wave at any azimuthal angle (blue arrows) is acquired, while the surface plasmonic wave is eliminated according to the formula. **d** Principle of azimuth-modulated plasmonic scattering interferometric microscopy. Only the objective-scattered surface plasmons (*E*_s_, pink arc) are acquired. **e** SEM and azimuth-modulated plasmonic scattering interferometric images of PS nanoparticles with various sizes (scale bars: 200 nm and 1 µm, respectively). **f** Calibration curve for a plot of plasmonic intensity versus radius fitted with Eq. 17. Error bars represent mean ± 2 standard deviation (*n*_r57.5_ = 243, *n*_r100_ = 380, *n*_r125_ = 377, *n*_r200_ = 273, *n*_r250_ = 49), and the centers of the error bars (black spot) represent mean values. Source data are provided as a Source Data file.

We designed an azimuth-modulated plasmonic scattering interferometric microscope based on the Kretschmann configuration^17,21^. To excite surface plasmonic waves, we directed light at an appropriate angle via an oil-immersion objective onto a gold-coated glass chip placed on the objective (Fig. 1a). Light reflected from the gold chip surface was then collected and imaged with a camera, as described by^22^:

1 $${I}_{{{{\rm{bg}}}}}={{{{\rm{|}}}}{E}_{{{{\rm{i}}}}}{{{\rm{|}}}}}^{2}={|{{{{\rm{e}}}}}^{{{{\rm{i}}}}{kr}}|}^{2}$$

where *I*_bg_ is the background intensity, *E*_i_ describes the excited incident surface plasmon *E*-field, *r* is the position, and *k* is the wave vector. The signal of an object in the field is described by:

2 $$I={|{E}_{{{{\rm{i}}}}}+{E}_{{{{\rm{s}}}}}|}^{2}$$

where *E*_s_ is the surface plasmon scattering field. The image is generated by interference between the objective-scattered surface plasmons *E*_s_ and *E*_i_ (Fig. 1b). To eliminate interference from reflected light on the scattered signal, we modulated the incident light by using a scanning galvanometer to rotate around the back focal plane (BFP) of the objective and form a circular orbit (red arrow in Fig. 1a). This modulation resulted in a surface plasmonic wave propagating on the Au surface at any azimuthal angle (red line in Fig. 1c, azimuthal angle *θ* from 0 to 2π). We synchronized the light modulation with camera sampling, which allowed each captured image to contain an azimuthal angle varying exactly from 0 to 2π. Therefore, the plasmonic image captured by azimuth-modulated plasmonic scattering interferometric microscopy can be described by:

3 $${{{\rm{d}}}}{I}_{{{{\rm{rot}}}}}(r)={{\int }_{\!\!\!\vartheta=0}^{\vartheta=2{{\uppi }}}}(I-{I}_{{{{\rm{bg}}}}}){{{\rm{d}}}}\vartheta={\int }_{\!\!\!\vartheta=0}^{\vartheta=2{{\uppi }}}{|{E}_{{{{\rm{i}}}}}+{E}_{{{{\rm{s}}}}}|}^{2}{{{\rm{d}}}}\vartheta - {\int }_{\vartheta=0}^{{\vartheta=2\pi }}{ | {E}_{{{{\rm{i}}}}} | }^{2}{{{\rm{d}}}}\vartheta $$

which is simplified as:

4 $${{{\rm{d}}}}{I}_{{{{\rm{rot}}}}}\left(r\right)=2{{\uppi }}\,\left[{|{E}_{{{{\rm{s}}}}}|}^{2}+2 \,{{{\rm{real}}}}({E}_{{{{\rm{s}}}}})\,{J}_{0}\left({r|k|}\right)\right]$$

where *J*_0_ is the Bessel function of the first kind. Detailed information about the formula derivation process can be found in the Methods section. *E*_s_ depends on the refractive index of the nanoparticle^19^, as follows:

5 $${E}_{{{{\rm{s}}}}}(r,\, \psi )=\beta \,{{\exp }}\left({{{\rm{i}}}}\psi \right)\,{{\exp }}\left(-\kappa \left|r\right|\right)\, {{\exp }}(-k{{{\rm{i|}}}}r{{|}})$$

where *β* is the scattering coefficient, *κ* is the decay constant of surface plasmons, and *ψ* is the phase of the scattering field, which offers extraordinary sensitivity to probe compositional changes at the nanoparticle, such as surface reactions. The simplified result of the formulation verifies that reflected light has been eliminated and that only the surface plasmonic interferometric scattering pattern is preserved in the image. Therefore, the plasmonic image can be described as a function of *ψ*:

6 $${{{\rm{d}}}}{I}_{{{{\rm{rot}}}}}(r)\propto {{\cos }}\,(\psi -{k|r|})$$

Consequently, this method avoids strong interference from reflected light and has an improved spatial resolution while avoiding interference from the parabolic-like interferometric pattern (Fig. 1d). Moreover, the intensity is tuned with *ψ*, which is a function of the refractive index of the nanoparticle, implying that the compositional changes can be probed with the images.

To experimentally validate the physical principles of plasmonic scattering interferometric imaging and explore the relationship between the plasmonic signal and the size of the object, we used polystyrene nanoparticles with standardized radii ranging from 57.5 to 250 nm. The nanoparticles were dispersed in a 20 mM KCl solution and introduced to the microreaction pool. Individual binding events were recorded with a charge-coupled device (CCD) camera operating at a rate of 50 frames per second (fps). As the evanescent field was confined to the near surface of the gold chip, this feature makes it immune to interference from nanoparticles and impurities in the bulk solution. Consequently, only the nanoparticles bound to the gold surface were imaged by the camera. With the scattering interference background suppressed by rotating the incident light, the representative images in Fig. 1e show a single bright spot with high contrast in the image. To verify the sizes of the polystyrene nanoparticles, scanning electron microscopy (SEM) was utilized for nanoparticle characterization (Supplementary Figs. 1–5). Figure 1e shows the SEM images and corresponding plasmonic images of polystyrene nanoparticles for comparison. The plasmonic intensity of the nanoparticles was highly dependent on their size (Fig. 1e). As a scattering-based imaging technique, the plasmonic intensity is theoretically correlated with the size and dielectric constant of the nanoparticle. We statistically calculated the plasmonic intensities of hundreds of single-sized nanoparticles and correlated the image intensities with nanoparticle sizes (Fig. 1f). Quantitative agreement was observed between the experimental results and the simulation results (orange line in Fig. 1f, the theoretical model is illustrated in Supplementary Fig. 8, details in Methods). Based on the noise level (Supplementary Fig. 9) and the above fitting curve (Fig. 1f), we analyzed the detection limit for a single polystyrene nanoparticle, which was approximately 28 nm in radius. Corresponding experimental evidence was provided to further support this limit (Supplementary Fig. 10). This could be improved further by noise reduction and signal processing. These results validated the controlling principle of plasmonic scattering interferometric imaging, implying a promising platform for nano-objective detection and imaging.

### Mapping the reactive region on a single Ag nanowire

**Fig. 2:**
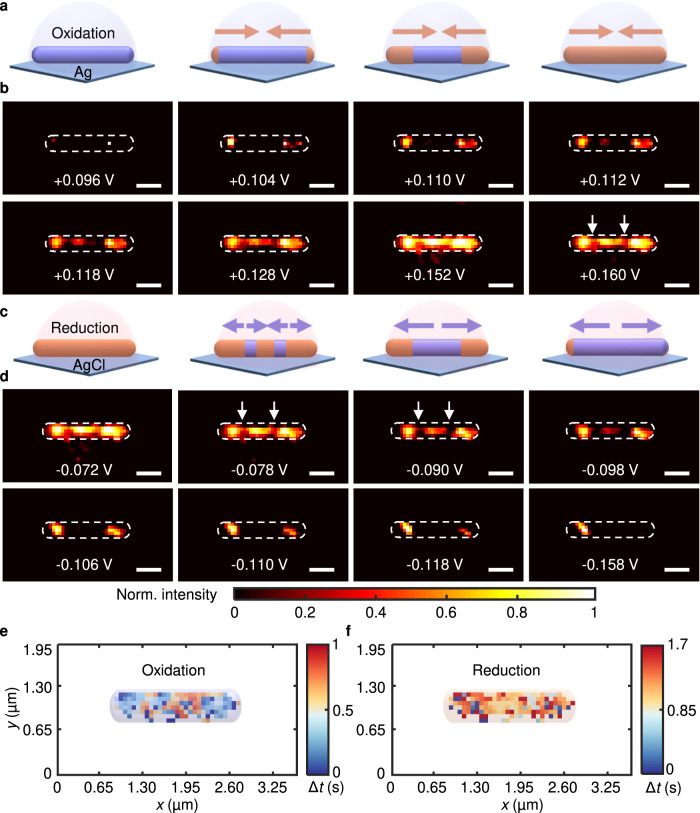
Plasmonic scattering interferometric mapping of the reactive site on a single Ag nanowire in KCl solution during the CV process. **a** Schematic of the oxidation process of a single Ag nanowire (purple) during the CV process. **b** Snapshot plasmonic images of a single Ag nanowire during the oxidation process (scale bar: 500 nm). **c** Schematic of the reduction process of a single AgCl nanowire (brown) during the CV process. **d** Snapshot plasmonic images of a single AgCl nanowire during the reduction process (scale bar: 500 nm). **e** Mapping of the original oxidation time delay on the nanowire. **f** Mapping of the original reduction time delay on the nanowire.

One of the primary benefits of our microscopy system is that the interference background can be suppressed under normal incidence without additional modulation in the Fourier plane^23^, thereby reducing the complexity of the imaging system and image processing and allowing for in situ chemical reaction monitoring. To demonstrate the capability for high-spatial resolution imaging of chemical reactions, we studied an electrochemical reaction over nanowires. Here, we used Ag nanowires as an example (Supplementary Fig. 11) since Ag-based nanomaterials are of interest for electrocatalytic/photocatalytic reactions^24,25^. Ag was converted to AgX in the presence of X^-^ during the electrochemical process occurring in a KX solution (Fig. 2a). We dispersed the Ag nanowires on the gold chip and employed this chip as the working electrode (a platinum wire served as the counter electrode and Ag/AgCl as the reference electrode) to construct a three-electrode electrochemical cell. The potential was swept between −0.4 V and +0.4 V for cyclic voltammetry (CV) studies in KCl solution, and the plasmonic images were recorded by a camera operating at 25 fps.

After subtracting the first frame, we localized and imaged the electrochemical reactions on the silver nanowire. A movie showing the electrochemical reaction is presented in the Supporting Information (Supplementary Movie 1). Figure 2 shows several snapshots from the movie at different potentials. As the positive scan potential reached approximately +0.096 V, we observed heterogeneous changes in the signal initialized from both ends of the nanowire, indicating oxidation of silver (Fig. 2b). The optical signal was propagated to the middle of the nanowire when the potential was further positively scanned, and finally, all sites on the nanowire became heterogeneously bright at a potential of +0.160 V (Fig. 2b). However, the optical intensities of the two regions were lower than those of other regions, as marked by the white arrows in Fig. 2b.

To map the reaction time, we developed a pixelwise reaction time estimation algorithm based on the Haar wavelet transform. Basically, this algorithm analyzed the time series of each pixel to determine the step change in intensity, which represented compositional variations occurring during electrochemical reactions. Reaction time delay mapping with this nanometer resolution further revealed that for the Ag nanowire, the surface reaction was heterogeneous, and the ends generally showed higher reactivity than the sides (Fig. 2e). Here, we used a fivefold twinned Ag nanowire, which had pentagonal cross-sections with fivefold twin longitudinal boundaries. The ends of this nanowire generally have a higher percentage of corner, twin boundary and edge sites, as evidenced by simulation results^26–28^ and transmission electronic microscopy images (Supplementary Fig. 12), and consequently higher reaction reactivity.

Interestingly, when the potential was swept negatively beyond −0.078 V, we noticed that changes began from the two regions marked with the white arrows in Fig. 2d, and sequentially, these signal variations propagated toward the ends of the nanowires as the potential was scanned to −0.4 V (Supplementary Movie 1, Fig. 2c, d). The signal from one location at the end did not disappear after the potential scan was finished (Fig. 2d), implying irreversibility of the electrochemical reduction at a certain location. Mapping of the reaction time delay further confirmed the above observations and revealed that the duration for reduction was longer than that for oxidation of Ag nanowires (Fig. 2f). The long duration indicated a slow reduction process, implying an intrinsic conductivity difference between Ag and AgCl. Furthermore, for some AgCl nanowires, we did not observe a reaction on the nanowire until the sixth pulse potential was applied, probably due to the electronic conductivity (Supplementary Fig. 13).

### High-throughput imaging of dynamics of single Ag nanowires

**Fig. 3:**
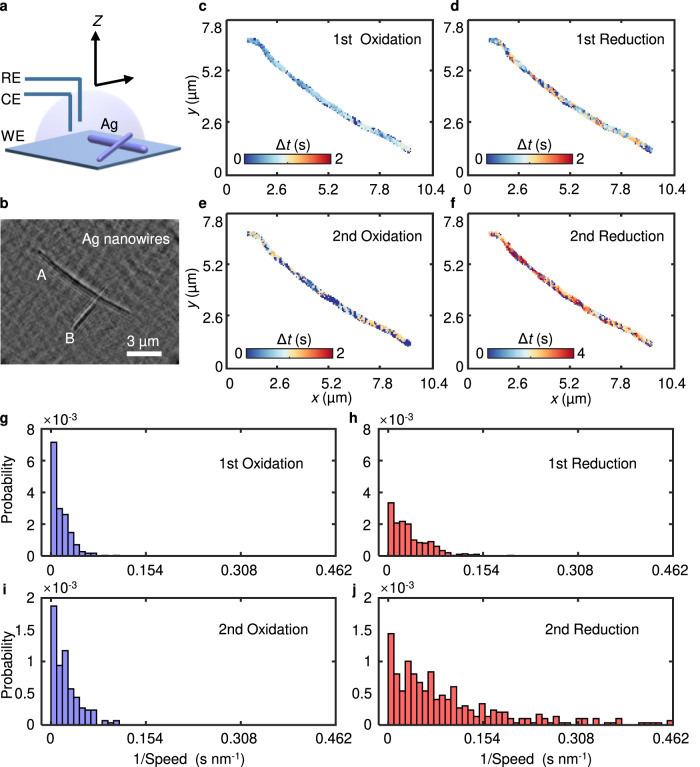
Plasmonic scattering interferometric imaging of interfacial dynamics with long Ag nanowires. **a** Schematic of two Ag nanowires (purple cylinders) on the chip during the CV process. RE (Reference electrode), CE (Counter electrode), WE (Working electrode). **b** Azimuth-modulated plasmonic scattering interferometric images of Ag nanowires (A and B, A is 10 µm, B is 4.3 µm). **c–f** Mapping of the original oxidation/reduction time delay of the first CV (1st Oxidation, 1st Reduction) and the second CV (2nd Oxidation, 2nd Reduction) along nanowire A. **g–j** Histogram of the oxidation/reduction speeds of nanowire A during the two CV cycles. Source data are provided as a Source Data file.

The wide-field imaging capability of our approach offers the possibility of studying hundreds of nanowires in parallel, giving high data throughput. Surprisingly, the relative reactivities of the ends and side of an Ag nanowire vary among individual nanowires, even though they share the same types of facets on their ends. Figure 3a, b show two longer nanowires piled up. There was no plasmonic intensity change observed on nanowire B while sweeping the potential (Supplementary Movie 2). This variant of the reactivity pattern was exhibited by a small population of nanowires and could be hidden in the ensemble-averaged results. To quantify the reactivity distribution on Nanowire A, we mapped the reaction time during CV processes (Fig. 3c–f). The oxidation reaction was initiated at several locations, including ends and sides on the nanowire, during the first positive scan, implying that these locations were more active (Fig. 3c). Occasionally, some nanowires have many defects and thus higher reactivities on their sides^29,30^. Atomic surface steps were found at the surface of the prepared Ag nanowires by transmission electron microscopy (TEM) (Supplementary Fig. 14). Furthermore, when we turned to the synthesis of nanowires, longer nanowires grew faster than shorter nanowires since they had more surface step defects on their sides^31–33^; this explains why the active locations were not confined only to the ends of Ag nanowires. We also statistically analyzed more nanowires. The results indicate that the region amount of initial oxidation on individual nanowires increased as the length of the nanowires increased, implying more surface step defects on longer nanowires (Supplementary Fig. 15). During the second positive scan, we observed a feature not seen during the first oxidation, and the distribution of the initial reaction time was random, implying that the electrochemical reaction not only allowed for relocating active sites but also transformed the morphology of the nanowire by redistributing surface atoms^34^. This result was confirmed by SEM images taken after the experiment (Supplementary Fig. 16). We also observed a difference between the first and second reduction processes (Fig. 3d, f).

To quantify the surface electrochemical dynamics for a single Ag nanowire, we statistically calculated the reaction propagation speeds during CV experiments. Figure 3g–j shows the distributions for reciprocals of propagating speeds. The distributions of reduction reactions were broader than those of oxidation processes, further confirming that the reduction of AgCl to Ag was slow and highly associated with electronic conduction. This information is completely hidden if a nanowire is analyzed as a single entity rather than by active region mapping, and it is only discoverable with higher spatial and real-time resolution of the reactivity of a single nanowire.

### Tracking electrochemical dissolution of a single Ag nanowire

**Fig. 4:**
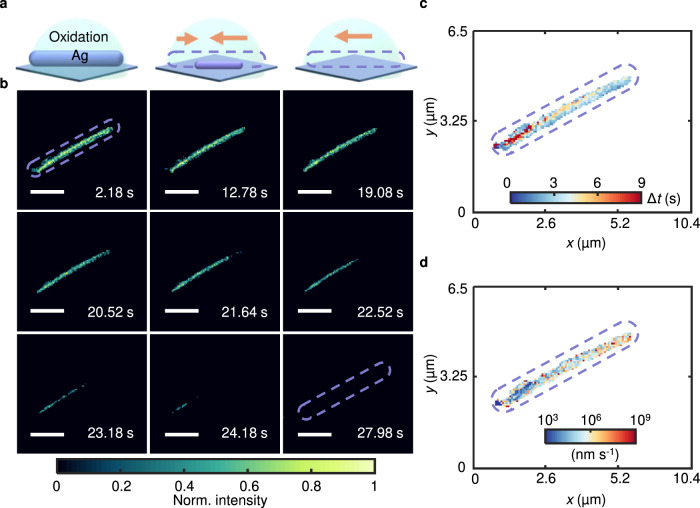
Azimuth-modulated plasmonic scattering interferometric imaging of the electrochemical dissolution of a single Ag nanowire in NaOH solution. **a** Schematic illustration of the experimental results. **b** Snapshot plasmonic images of Ag nanowire dissolution during the LSV process. **c** Mapping of the time delay of dissolution on the nanowire. **d** Mapping of the dissolution rate on the nanowire.

Based on the ability to achieve imaging of active sites, we demonstrated the use of this technique in probing the electrochemical reaction dynamics of a single nanowire in an alkaline electrolyte, which is an important process for single-entity electrocatalysis and yet is not fully understood^35–37^. This kind of reaction is of practical interest for studying the mechanisms of dissolution and oxide formation relevant to corrosion and catalysis^38,39^. We performed linear sweep voltammetry on silver nanowires in 100 mM NaOH solutions (Fig. 4a). Surprisingly, we observed gradual disappearance of the nanowire as the potential was scanned in the positive direction, which differed completely from the results obtained in KCl solution (Fig. 4b and Supplementary Movie 3). The accordance between the oxidation peak and the plasmonic intensity confirmed the electrochemical dissolution of the nanowire (Supplementary Fig. 17). The oxidation was attributed to the formation of Ag(I) oxides according to the following reactions:

7 $$2 \,{{{\rm{Ag}}}}+2 \,{{{\rm{O}}}}{{{{\rm{H}}}}}^{-}- 2 \,{{{{\rm{e}}}}}^{-}\to 2 \,{{{\rm{AgOH}}}}$$

8 $${2 \, {{{\rm{AgOH}}}}\to {{{\rm{Ag}}}}}_{2}{{{\rm{O}}}}+{{{{\rm{H}}}}}_{2}{{{\rm{O}}}}$$

Competitive dissolution and oxide formation in alkaline electrolytes have been confirmed by in situ electrochemical scanning tunneling microscopy (EC-STM)^40^. Nanoscale observations indicated that most of the Ag atoms were ejected from the surface by surface reconstruction, resulting in dissolution of the Ag nanowires^41^.

To quantify the dissolution dynamics, we mapped the initial dissolution time delay over the nanowire. The sites with larger time delays were almost completely confined to the interior of the nanowire (Fig. 4c), consistent with the above observation that dissolution occurred from external to internal in the radial direction and initiated from the ends in the axial direction (Fig. 4b). The reaction rate was highly dependent on the reactivities of the active sites. To compare the site-by-site reaction rates, we estimated the dissolution rate by calculating the gradient of the dissolution time (Fig. 4d). Unlike other subparticle-resolution imaging techniques, such as fluorescence single-molecule imaging^32^, which studies the reaction rate by counting reaction events over a long time and comparing different segments of the nanowire, the increase in the spatial resolution and real-time imaging capability of our method enabled us to visualize the reaction rate along a single nanowire. The sites with high dissolution rates were almost in full accordance with the reaction time delay map (Fig. 4c).

## Discussion

The spatial resolution of our method is lower than other AFM-, SEM- and TEM-based approaches due to the diffraction limits of the optical system, which are influenced by several factors, such as refractive-index of material, light source and image stacks for reconstruction. We developed a pixelwise reaction time estimation method to subsequently analyze the image stacks, which is independent of diffraction limits. Therefore, the spatial resolution can be improved by integrating advanced super-resolution imaging approaches. Besides, our method inevitably suffers from focus drift during long-time recordings due to the fluidity of objective oil, potentially affecting the accurate quantification of images. We therefore used a focus compensation system to reduce focus drift. More importantly, the reaction was fast, and our algorithm analyzed differential images, rendering drift-induced noise negligible in this situation.

In summary, we have developed an azimuth-modulated plasmonic imaging method and demonstrated its application to image dynamic interfacial changes in a single nanowire during chemical reactions. This work offers a widely applicable imaging strategy that addresses a central shortcoming of plasmonic imaging, namely, the impractical high spatial resolution imaging of surface chemical dynamics. The real-time observation of heterogeneous interfacial dynamics or transient structures could prove invaluable for the development of nanotechnology that critically relies on local structural and dynamical processes but is also useful for heterogeneous catalysis and electrochemistry. Although a gold-coated chip was used as the substrate, we demonstrated here that this technique could be used to answer some potential fundamental questions on surface electrochemical reactions. More importantly, gold substrates can also be used as electrodes to control the reaction potential. Since our technique provides insights into the heterogeneous reactivities of nanomaterials, we anticipate that it will offer even more information on surface chemistry and reactions at the nanoscale by integrating existing electron microscopy and synchrotron-based methodologies to characterize the structures and chemical composition of nanomaterials.

## Methods

### Materials

Polystyrene nanoparticles (5 wt.% solid content) with standardized radii of 57.5, 100, 125, 200, and 250 nm were purchased from Janus New-Materials Co., Ltd., China. KCl (≥99.5%) and NaOH (≥96%) were purchased from Sinopharm Chemical Reagent Co., Ltd. All reagents were directly used without further purification. The deionized water (a resistivity of 18.2 MΩ cm^−1^) used in the experiments was supplied from a deionized water system (Milli-Q, Millipore).

Ag nanowires were synthesized using the following experimental procedures: silver nitrate aqueous solution (5 mL, 0.5 M) was mixed with sodium chloride aqueous solution (5 mL, 1 M) under stirring at 800 rpm for 1 min. Upon NaCl addition, AgCl immediately flocculated, which was used for the next step. In a typical synthesis, 0.34 g of PVP (*M*_w_ 40,000 g mol^−1^) was dissolved into 20 mL of ethylene glycol in a 25 mL flask and heated to 160 °C while stirring at 800 rpm. Once the solution had reached a stable temperature, an excess (25 mg) of freshly prepared AgCl was added all at once, and the solution turned from transparent to light yellow. After 3 min, 0.11 g of AgNO_3_ was added to the above solution, and the round-bottom flask was sealed with a septum cap. The reaction mixture was stirred at 160 °C for 24 min. Finally, Ag nanowires were prepared. The obtained product was centrifuged at 8603 *×* *g* for 10 min and then washed with deionized water three times before the subsequent experiments.

### Characterization

SEM (Sirion 200, FEI Electron Optics Co., USA) was used to characterize the samples. XRD patterns of the products were recorded on a Philips X’pert X-ray diffractometer with Cu Ka radiation (λ = 1.54182 Å) at a scan rate of 0.008842° s^−1^. HRTEM (JEM-F200, JEOL, Japan) was used to image Ag nanowires.

### Setup of azimuth-modulated plasmonic scattering interferometric microscopy

The plasmonic microscope was based on a homemade total inverted microscope with a high-numerical-aperture oil-immersion objective (100×, NA = 1.49). Incident light from a 670 nm superluminescent diode (Qphotonics, USA) was directed via the objective onto a coverslip coated with a gold layer (thickness 47 nm) to excite the surface plasmonic wave. A scanning galvanometer (GVS012, Thorlabs, USA) was used to modulate the location of the incident light on the BFP of the objective. The scattering interferometric light was imaged by a CCD camera (Pike F-032B, Allied Vision Technologies, Germany). The light swept along a circular orbit at a rotation speed of 10 ms per revolution (100 rad s^−1^) under the modulation of the galvanometer. To synchronize with the light sweeping frequency, the frame rate of the camera was set at 25 or 50 fps, which allowed the image acquisition of each frame containing the signal along the azimuthal angle exactly from 0 to 2π. Since the reflected light from all azimuths canceled each other out within the sampling period, the integral image of the object achieves an improved spatial resolution.

### Plasmonic image formation

The plasmonic image is formed by the interference of the plane wave (*E*_p_) and the scattered wave (*E*_s_):

9 $$I\left(r,\, \theta \right) 	=\left({E}_{{{{\rm{p}}}}}\left(r,\, \theta \right)+{E}_{{{{\rm{s}}}}}(r)\right)\, * \, ({E}_{{{{\rm{sp}}}}}^{ * }\left(r,\,\theta \right)+{E}_{{{{\rm{s}}}}}^{ * }(r)) \\ 	={\left|{E}_{{{{\rm{p}}}}}\left(r,\, \theta \right)\right|}^{2}+{{{{\rm{|}}}}{E}_{{{{\rm{s}}}}}{{{\rm{|}}}}}^{2}+{E}_{{{{\rm{p}}}}}\left(r,\, \theta \right)\, * \, {E}_{{{{\rm{s}}}}}^{ * }\left(r\right){+E}_{{{{\rm{sp}}}}}^{*}\left(r,\, \theta \right)\, * \, {E}_{{{{\rm{s}}}}}(r)$$

where *r* represents a spherical coordinate and *θ* is the propagation direction of the plane wave. Since the anisotropy of the scattered light is not taken into account, *E*_s_ is not related to *θ*.

The image after rotation of the plane wave can be expressed as:

10 $${I}_{{{{\rm{rot}}}}}(r)=	 {\int }_{\!\!\!\!0}^{2{{\uppi }}}I\left(r,\, \theta \right){{{\rm{d}}}}{{\alpha }}={\int }_{\!\!\!\!0}^{2{{\uppi }}}{\left|{E}_{{{{\rm{p}}}}}\left(r,\,\theta \right)\right|}^{2}{{{\rm{d}}}}\theta+2{{\uppi }}{\left|{E}_{{{{\rm{s}}}}}\left(r\right)\right|}^{2}\\ 	+{\int }_{\!\!\!\!0}^{2{{\uppi }}}{E}_{{{{\rm{p}}}}}\left(r,\, \theta \right)\, * \,{E}_{{{{\rm{s}}}}}^{ * }\left(r\right){+E}_{{{{\rm{p}}}}}^{ * }\left(r,\, \theta \right) * {E}_{{{{\rm{s}}}}}\left(r\right){{{\rm{d}}}}\theta $$

Here, ${\left|{E}_{{{{\rm{p}}}}}\left(r,\theta \right)\right|}^{2}$ is the background if we remove the background:

11 $${{{\rm{d}}}}{I}_{{{{\rm{rot}}}}}\left(r\right)=	 {\int }_{\!\!\!\!0}^{2{{\uppi }}}\left(I\left(r,\, \theta \right)-{I}_{{{{\rm{bg}}}}}\right)d\theta \\ =	2{{\uppi }}{\left | {E}_{{{{\rm{s}}}}}\left(r \right) \right | }^{2}+ {\int }_{\!\!\!\!0}^{2{{\uppi }}}({E}_{{{{\rm{p}}}}}\left(r,\, \theta \right)\, * \, {E}_{{{{\rm{s}}}}}^{ * }\left(r \right){+ E}_{{{{\rm{p}}}}}^{ * }\left(r,\, \theta \right)\, * \,{E}_{{{{\rm{s}}}}}\left(r\right)){{{\rm{d}}}} \theta$$

Furthermore, the electric field of the plane wave and the scattered wave can be expressed as:

12 $${E}_{{{\rm{p}}}}(r,\, \theta )={{{{\rm{e}}}}}^{{{{\rm{i}}}}P(r,\, \theta )}$$

13 $${{E}_{{{\rm{s}}}}} {\left(r\right)}={{S}_{1}} {(r)} {{e}^ {{{{\rm{i}}}} {\ast} {{S}_{2}}}} (r,\, \psi) $$

where *ψ* is the phase-dependent term, *S*_1_ is the relative amplitude to the plane wave, and *P* and *S*_2_ are the arguments of the plane wave and the scattered wave, respectively.

14 $${\int }_{\!\!\!\!0}^{2{{\uppi }}}	({E}_{{{{\rm{p}}}}}\left(r,\, \theta \right)\, * \,{E}_{{{{\rm{s}}}}}^{ * }\left(r\right){+E}_{{{{\rm{p}}}}}^{ * }\left(r, \theta \right)\, * \,{E}_{{{{\rm{s}}}}}\left(r\right)){{{\rm{d}}}}\theta \\ 	={\int }_{\!\!\!\!0}^{2{{\uppi }}}{S}_{1}\left(r\right)({e}^{{{{\rm{i}}}}\left(P\left(r, \theta \right)-{S}_{2}\left(r \,, \psi \right)\right)}+{{{{\rm{e}}}}}^{{{{\rm{i}}}}\left({S}_{2}\left(r,\, \psi \right)-P\left(r,\, \theta \right)\right)}){{{\rm{d}}}} \theta \\ 	={S}_{1}\left(r\right){\int }_{\!\!\!\!0}^{2{{\uppi }}}({{{{\rm{e}}}}}^{{{{\rm{i}}}}\left(P\left(r,\, \theta \right)-{S}_{2}\left(r,\, \psi \right)\right)}+{{{{\rm{e}}}}}^{{{{\rm{i}}}}\left({S}_{2}\left(r,\, \psi \right)-P\left(r,\, \theta \right)\right)}){{{\rm{d}}}}\theta \\ 	=2\,{S}_{1}\left(r\right)\,{{\cos }}\left({S}_{2}\left(r,\, \psi \right)\right){\int }_{\!\!\!\!0}^{2{{\uppi }}}{{\cos }}\left(P\left(r,\, \theta \right)\right){{{\rm{d}}}}\theta \\ 	\quad+2\,{S}_{1}\left(r\right)\,{{\sin }}\left({S}_{2}\left(r,\, \psi \right)\right){\int }_{\!\!\!\!0}^{2{{\uppi }}}{{\sin }}\left(P\left(r,\, \theta \right)\right){{{\rm{d}}}}\theta $$

In Cartesian coordinates, *P* can be written as:

15 $$P \left (r,\, \theta \right)=k ({{x}\,{\cos}({\theta}) {+} {y}\,{\sin}({\theta})})$$

where *k* is the wave vector. Finally, we can obtain:

16 $${{{\rm{d}}}}{I}_{{{{\rm{rot}}}}}\left(r\right)=2{{\uppi }}({\left|{E}_{{{{\rm{s}}}}}\right |}^{2}+2 \,{{{\rm{real}}}}\left({E}_{{{{\rm{s}}}}}\right)\,{J}_{0}\left(r\left|k\right | \right))$$

where *J*_0_ is the Bessel function of the first kind.

### Numerical model of plasmonic scattering interferometric intensity versus particle size

Considering that the excited surface plasmon *E*-field *E*_i_ decays exponentially with the distance from the surface plasmon, the intensity is expressed as^23,42^:

17 $$I=C+k{\int }_{0}^{2r}{{\uppi }}\left(2{rz}-{z}^{2}\right){{{{\rm{e}}}}}^{-z/l}{{{\rm{d}}}}z$$

where *C* and *k* are fitted constants, *z* is the distance above the surface, *r* is the radius of the particle, and *l* is the decay constant of the evanescent field, which is approximately 200 nm.

### Spatial resolution of azimuth-modulated plasmonic scattering interferometric microscopy

The spatial resolution was evaluated by calculating the value of full width at half maximum (FWHM) of the intensity profile across the nanoparticle. For our method, the achieved spatial resolution is approximately 300 nm FWHM in both longitudinal and transverse directions (Supplementary Figs. 6, 7, see Supplementary Note 1 for details), which is close to the diffraction limit of the optical system (315 nm).

### Detection limit

We examined collisions of polystyrene particles with different sizes on the sensing surface and analyzed the noise level in our setup. The signal-to-noise ratio (SNR) was defined as the difference between the signal and average background intensity divided by the standard deviation of the background noise. The plasmonic image intensity of single polystyrene particles was calculated as the mean intensity of a 4 × 4-pixel area (region of interest) around the spot center (left insets) after subtracting the background intensity. The standard deviation of the noise of the background image was 1.51 × 10^4^. We defined three times the SNR as the detection limit. According to the noise level and the fitting curve of intensity versus nanoparticle size (Fig. 1f), we concluded that the detection limit for polystyrene nanoparticles is approximately 28 nm in radius. This could be further improved by noise reduction and signal processing.

### Surface electrochemical reaction imaging

To image the surface chemical reactions occurring on single Ag nanowires, the surface electrochemical reaction was taken as an example. An Au chip was used as the working electrode, and an Ag/AgCl wire and a Pt wire were used as the reference electrode and counter electrode, respectively. An electrochemical chamber (0.8 cm × 0.8 cm × 0.9 cm) made from polydimethylsiloxane (PDMS) was placed on top of the gold chip (2.2 cm × 2.2 cm) to hold the electrolyte (Supplementary Fig. 18). A potentiostat (CHI760E, Shanghai Chenhua, China) was used to control the electrical potential. IR-compensation was not performed. After Ag nanowires were dispersed on the Au chip, we started the electrochemical experiments. Plasmonic scattering interferometric images arising during the electrochemical processes were recorded by the camera at a frame rate of 25 or 50 fps.

Before imaging the electrochemical test, the prepared Ag nanowire stock solution was diluted to a suitable concentration. Then, 20 μL of diluted solution containing Ag nanowires was added to the cell. After Ag nanowires were dispersed on the gold layer (effective electrode area: 0.8 cm × 0.8 cm), 500 µL of NaBH_4_ solution (1 wt.%) was added to the cell to eliminate the PVP on the nanowires since NaBH_4_ treatment can readily remove PVP ligands from the Ag nanowire surface, which can ensure good electrical conductivity^43^. To image the electrochemical process of Ag nanowires in KCl solution, we added KCl solution (600 μL, 20 mM) into the PDMS cell. We then performed cyclic voltammetry (CV) in the range of −0.4 V and +0.4 V in KCl solution (scan rate: 0.05 V s^−1^). The plasmonic images were recorded by the camera at 25 fps for subsequent data processing. To image the electrochemical dissolution of Ag nanowires in NaOH solution, we switched the KCl solution to NaOH solution (600 μL, 100 mM) and performed linear sweep voltammetry (LSV) from −0.4 V to +0.4 V (scan rate: 0.05 V s^−1^). Experiments with Ag nanowires in a KBr solution were performed and the corresponding conversion process can also be imaged by our method (see Supplementary Fig. 19 for details).

### Data processing

Data processing was performed with MATLAB (2019b, MathWorks) software. We subtracted the average intensity of the area without nanowires from the image sequence to eliminate the intensity fluctuations induced by the charging effect and the random noise of the light source. To determine the turning point of the surface reaction on the nanowire, continuous wavelet transformation (CWT) with the Haar wavelet was employed to analyze the time series of each pixel, which can be described as:

18 $${h}_{a,b}\left(t\right)=\frac{1}{\sqrt{a}}h\left(\frac{t-b}{a}\right)$$

where *a* and *b* are the scaling and shifting factors, respectively. The Haar wavelet (*h*) is:

19 $$h(t)=\left\{ \begin{array}{cc}1 & 0\,\le \, t\, < \, 1/2,\\ -1 & 1/2\, \le \, t\, < \, 1,\\ 0 & {{{\rm{otherwise}}}}.\end{array}\right.$$

The CWT maps the time series of the image *f (t)* onto time-scale space by:

20 $${{WT}}_{f}\left(a,\, b\right)=\left\langle \,{f\left(t\right),\, h}_{a,\, b}\left(t\right)\right\rangle =\frac{1}{\sqrt{a}}{\int }_{\!\!\!\!-{{{\rm{\infty }}}}}^{+{{\infty }}}f(t)\, h\left(\frac{t-b}{a}\right){{{\rm{d}}}}t.$$

where the shifting factor *b* indicates the turning point for Ag oxidation/AgCl reduction. For example, a time series of a pixel from the Ag nanowire during CV in the range of −0.4 V and +0.4 V is shown in Supplementary Fig. 20a. The time-scale space mapped by CWT indicates that the Ag oxidation/AgCl reduction resulted in a local maxima/minima of *b* (Supplementary Fig. 20b). The scaling factor was selected as 100 in our experiment. To estimate the time resolution of our method, we simulated a signal pixel time series with different SNRs and transformation times using the Monte Carlo method (Supplementary Fig. 21). Compared with the data acquired from the experiment (red point in Supplementary Fig. 21), the average length of the confidence intervals of the turning point is 11 ms. The gradient of the turning point (${{{\rm{d}}}}t/{{{\rm{d}}}}x$, ${{{\rm{d}}}}t/{{{\rm{d}}}}y$ for the *x* and *y* directions, respectively) indicated the interval time of the reaction between two adjacent pixels, while the spread speed of the reaction was given as $\sqrt{{({{{\rm{d}}}}x/{{{\rm{d}}}}t)}^{2}+{({{{\rm{d}}}}y/{{{\rm{d}}}}t)}^{2}}$.

## Supplementary information

Supplementary Information

Description of Additional Supplementary Information

Supplementary Movie 1

Supplementary Movie 2

Supplementary Movie 3

## Data Availability

The data that support the findings of this study are available from the corresponding authors upon request. Source data are provided with this paper.

## Code availability

The code for the image processing is available via GitHub and Zenodo^44^.
