# Supplementary material for: Dynamic imaging of interfacial electrochemistry on single Ag nanowires by azimuth-modulated plasmonic scattering interferometry
Source: Nat Commun. 2023 Jul 13;14:4194. doi: 10.1038/s41467-023-39866-8 (PMC10344930; doi:10.1038/s41467-023-39866-8)
Supplement: Supplementary file 1 — Supplementary Information [file 41467_2023_39866_MOESM1_ESM.pdf]

Supplementary Information for

Dynamic imaging of interfacial electrochemistry on single Ag nanowires by azimuth-modulated plasmonic scattering interferometry

Gang Wu[1], Chen Qian[1*], Wen-Li Lv[1], Xiaona Zhao[1], and Xian-Wei Liu[1,2*]

[1]Chinese Academy of Sciences Key Laboratory of Urban Pollutant Conversion, Department of Environmental Science and Engineering, University of Science and Technology of China, Hefei, 230026, China

[2]Department of Applied Chemistry, University of Science and Technology of China, Hefei, 230026, China

*Correspondence to: qianc@ustc.edu.cn (C Qian) or xianweiliu@ustc.edu.cn (XW Liu)

Table of Contents

Supplementary Figures 3

1.1 Morphology of polystyrene nanoparticles with different diameters 3

1.2 The spatial resolution of azimuth-modulated plasmonic scattering interferometric microscopy 7

Supplementary note 1...8

1.3 Numerical model of plasmonic scattering interferometric intensity versus particle size and detection Limit 9

Supplementary note 2...11

1.4 Surface morphology and chemical composition of Ag nanowires.....................12

Supplementary note 3...13

1.5 Electrochemical reaction process of a AgCl nanowire 15

1.6 Penta-twinned Ag nanowires...16

1.7 Electrochemical reaction process of single Ag nanowires of different lengths .17

1.8 Characterization of Ag nanowires before/after reaction 18

1.9 Tracking the electrochemical dissolution process of a single Ag nanowire 19

1.10 Scheme of the electrochemical chamber..20

1.11 Tracking the electrochemical process of single Ag nanowires in KBr solution 21

1.12 Data processing 22

Supplementary Figures

1.1 Morphology of polystyrene nanoparticles with different diameters

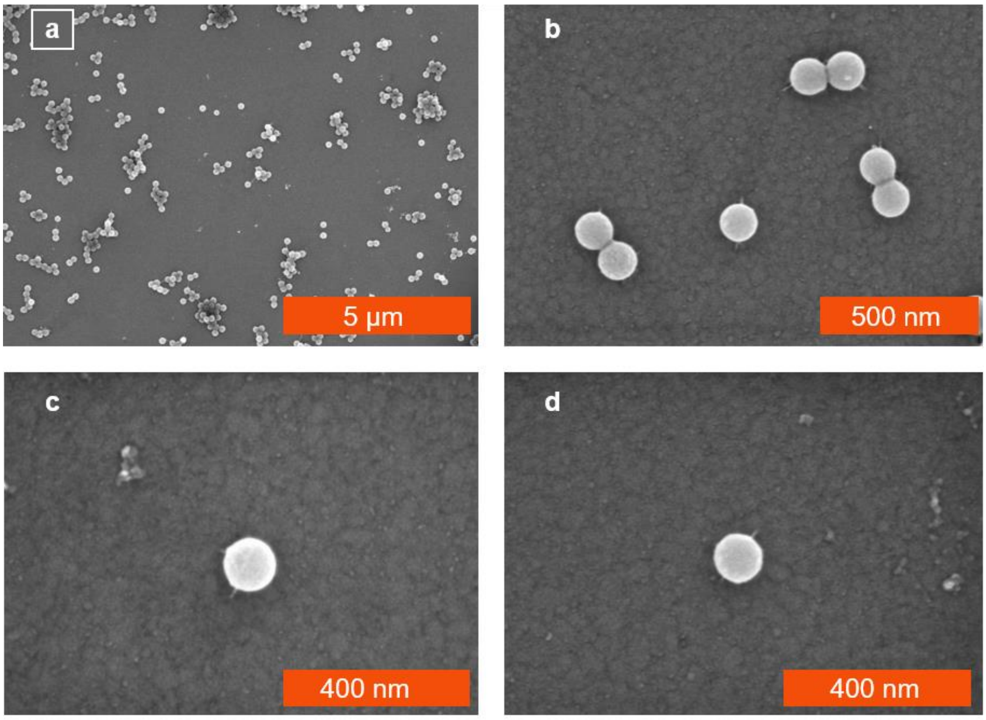

Supplementary Fig. 1. Morphology of 115 nm polystyrene nanoparticles. (a-d) SEM image of 115 nm polystyrene nanoparticles. Scale bar: (a) 5 μm; (b) 500 nm; (c, d) 400 nm.

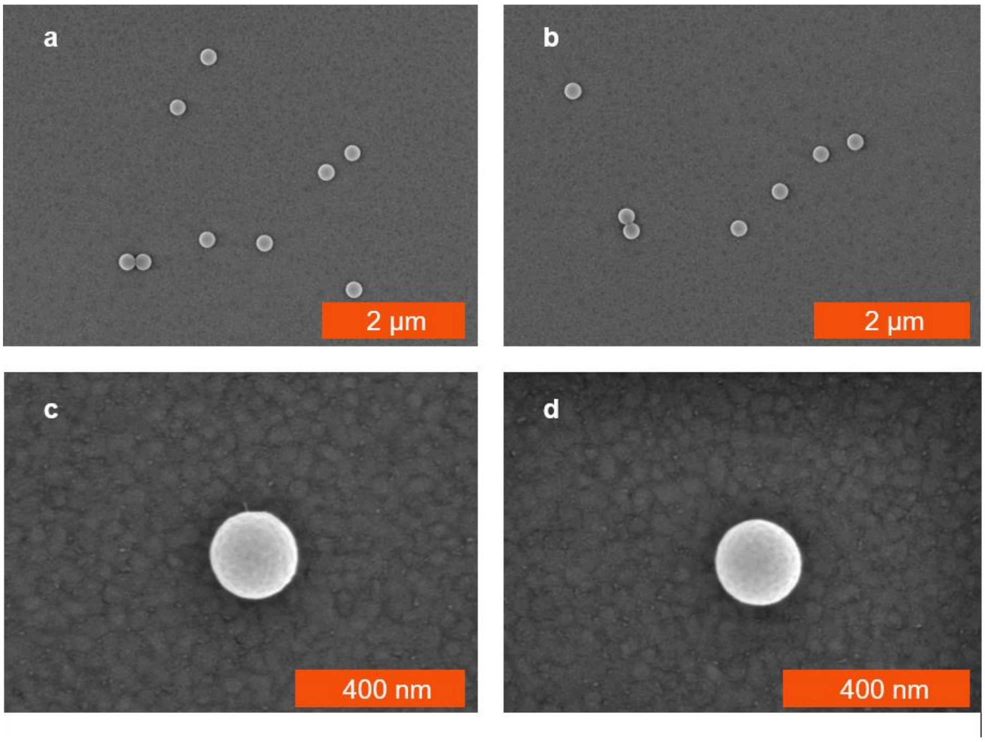

Supplementary Fig. 2. Morphology of 200 nm polystyrene nanoparticles. (a-d) SEM image of 200 nm polystyrene nanoparticles. Scale bar: (a, b) 2 μm; (c, d) 400 nm.

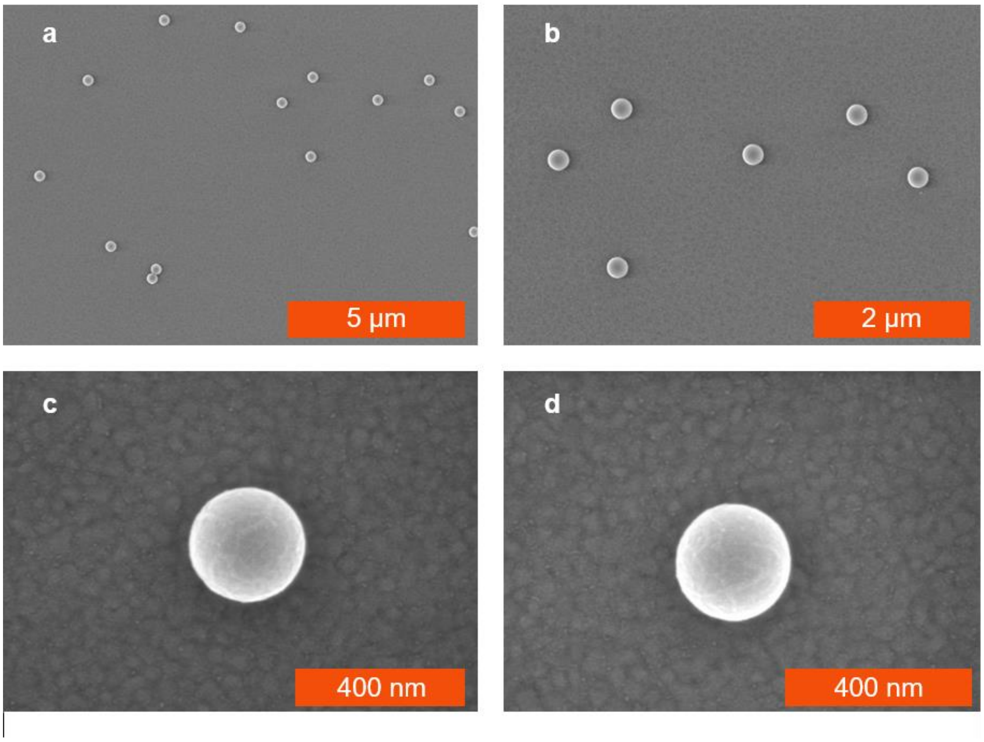

Supplementary Fig. 3. Morphology of 250 nm polystyrene nanoparticles. (a-d) SEM image of 250 nm polystyrene nanoparticles. Scale bar: (a) 5 μm; (b) 2 μm; (c, d) 400 nm.

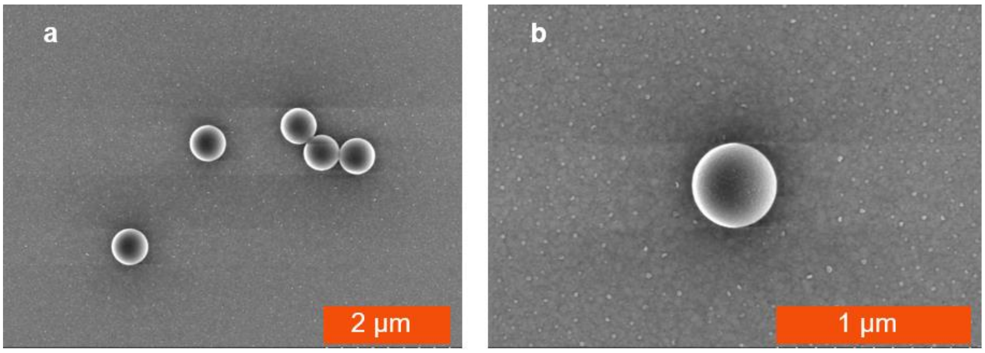

Supplementary Fig. 4. Morphology of 400 nm polystyrene nanoparticles. (a, b) SEM image of 400 nm polystyrene nanoparticles. Scale bar: (a) 2 μm; (b) 1 μm.

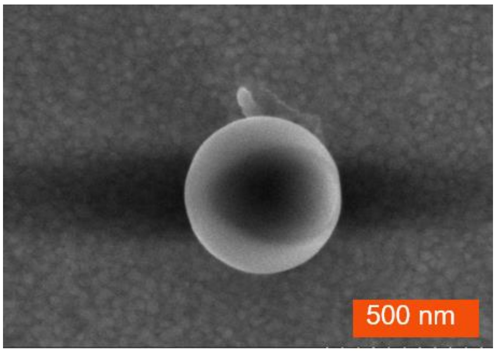

Supplementary Fig. 5. Morphology of 500 nm polystyrene nanoparticles. SEM image of 500 nm polystyrene nanoparticles. Scale bar: 500 nm.

1.2 The spatial resolution of azimuth-modulated plasmonic scattering interferometric microscopy

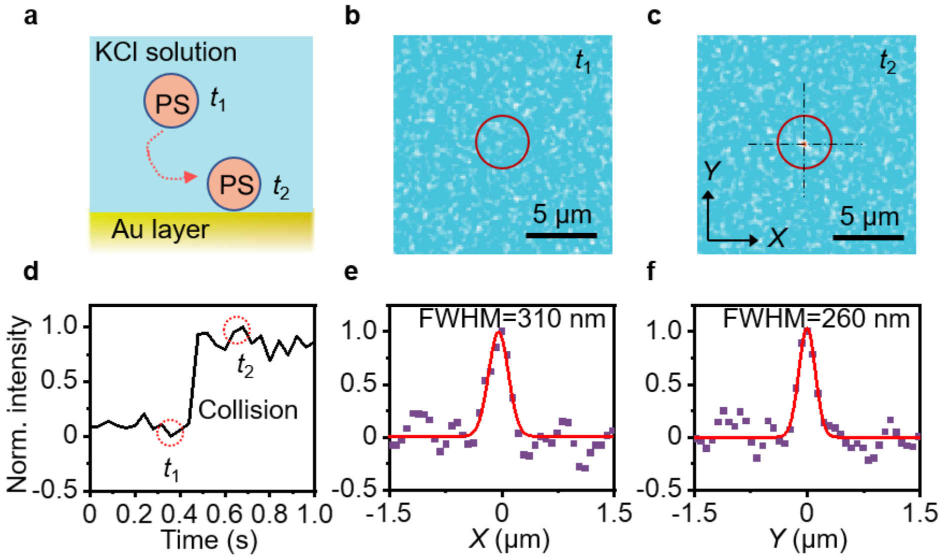

Supplementary Fig. 6. The spatial resolution of azimuth-modulated plasmonic scattering interferometric microscopy was determined with single PS nanoparticles. The collision of a single PS nanoparticle is schematically illustrated in (a). Azimuth-modulated plasmonic scattering interferometric images were captured at two different times: (b) during the absence of PS nanoparticles at t_1 and (c) during the presence of a PS nanoparticle (radius=25 nm) on the Au layer at t_2. The intensity variations during the collision process of the single PS nanoparticle are plotted in (d). The intensity profile across the nanoparticle marked in (c) by the black dashed lines in the X direction is shown in (e), while the intensity profile in the Y direction is displayed in (f).

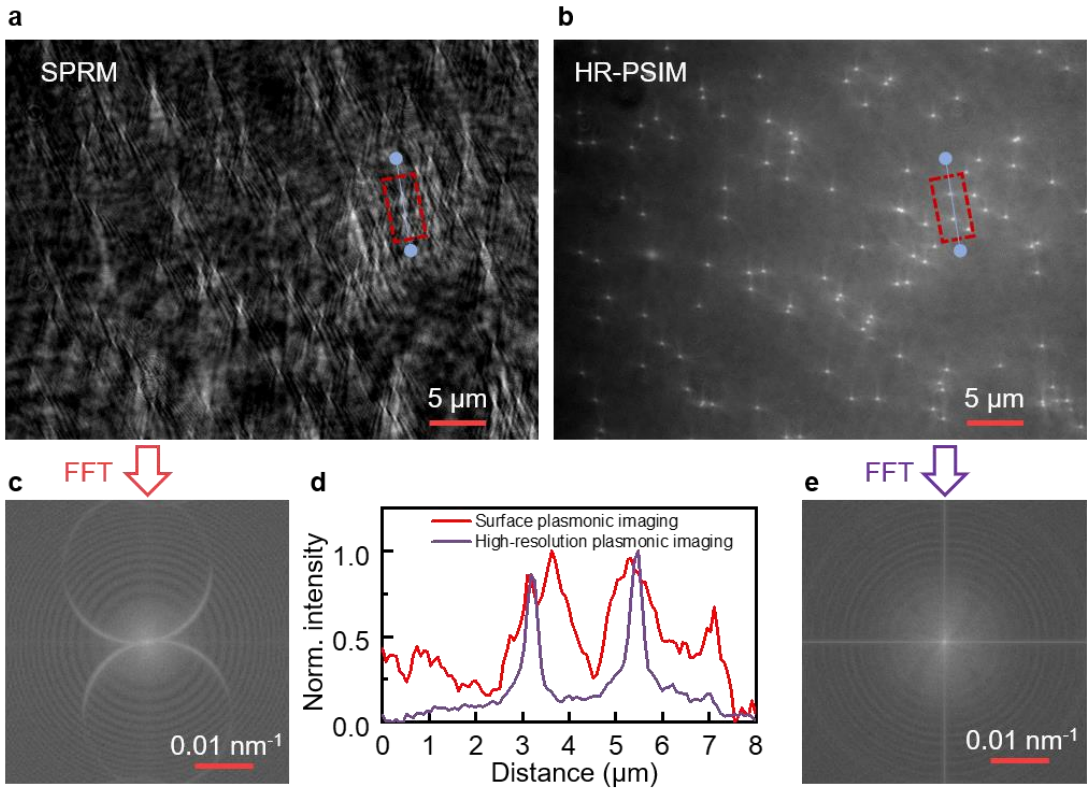

Supplementary Fig. 7. Comparison of SPRM (surface plasmonic resonance microscopy) with HR-PSIM (high-resolution plasmonic scattering interferometric microscopy). (a) Traditional plasmonic images and (b) azimuth-modulated plasmonic scattering interferometric image of single polystyrene nanoparticles (radius=200 nm) and corresponding Fourier space image (c) and (e). (d) Intensity profile across the two nanoparticles marked in (a) and (b) by the blue solid lines.

Supplementary note 1: Figure S7 compares traditional plasmonic microscopy and our technique for imaging 200 nm nanoparticles with the same field of view. The spatial resolution of plasmonic imaging has significantly increased by 67-fold from the previous micrometer (20 µm) to the nanometer scale (300 nm). The Fourier space image of the azimuth-modulated plasmonic scattering interferometric image shows no ring-like feature, indicating the elimination of strong interference from the interferometric pattern.

1.3 Numerical model of plasmonic scattering interferometric intensity versus particle size and detection Limit

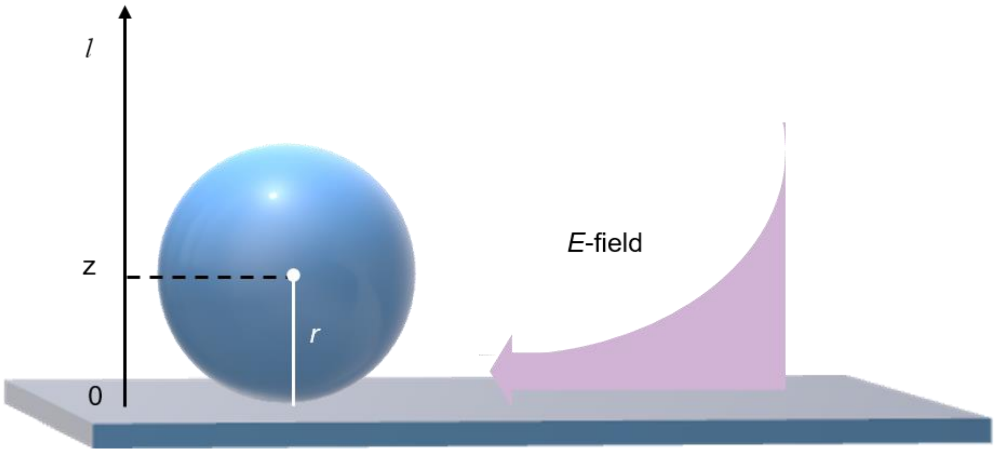

Supplementary Fig. 8. Schematic diagram of numerical model. Theoretical model of plasmonic scattering interferometric intensity versus particle size.

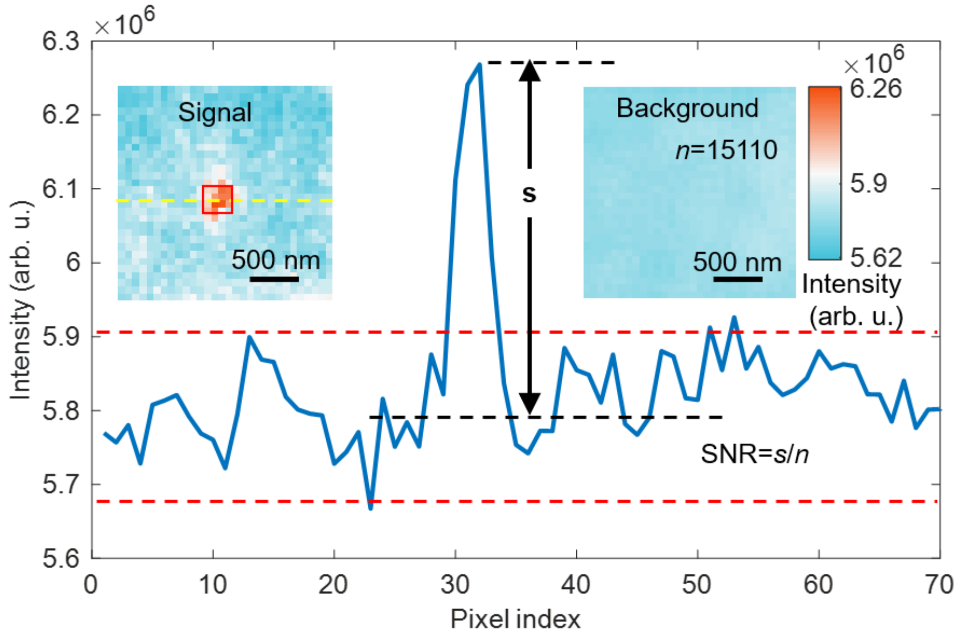

Supplementary Fig. 9. Schematic diagram of the signal-to-noise ratio (SNR). SNR and image intensity in the plasmonic scattering interferometric image, where s is the signal intensity and n is the standard deviation of the background noise.

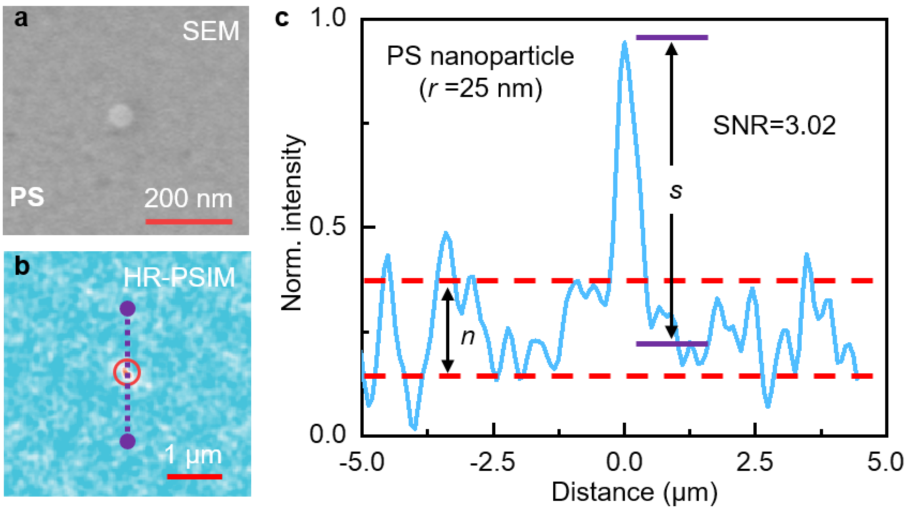

Supplementary Fig. 10. The detection limit of azimuth-modulated plasmonic scattering interferometric microscopy for a single polystyrene nanoparticle. Images of a single PS nanoparticle with a radius of 25 nm were captured using SEM (a) and azimuth-modulated plasmonic scattering interferometric microscopy (b). The intensity profile across the nanoparticles marked in (b) by the dashed lines is shown in (c), where s represents the signal intensity and n represents the standard deviation of the background noise.

Supplementary note 2: Single polystyrene nanoparticles with a radius of 25 nm on the chip were imaged, and the signal-to-noise ratio was 3.02, which is consistent with our predicted result (Supplementary Fig. 10).

1.4 Surface morphology and chemical composition of Ag nanowires

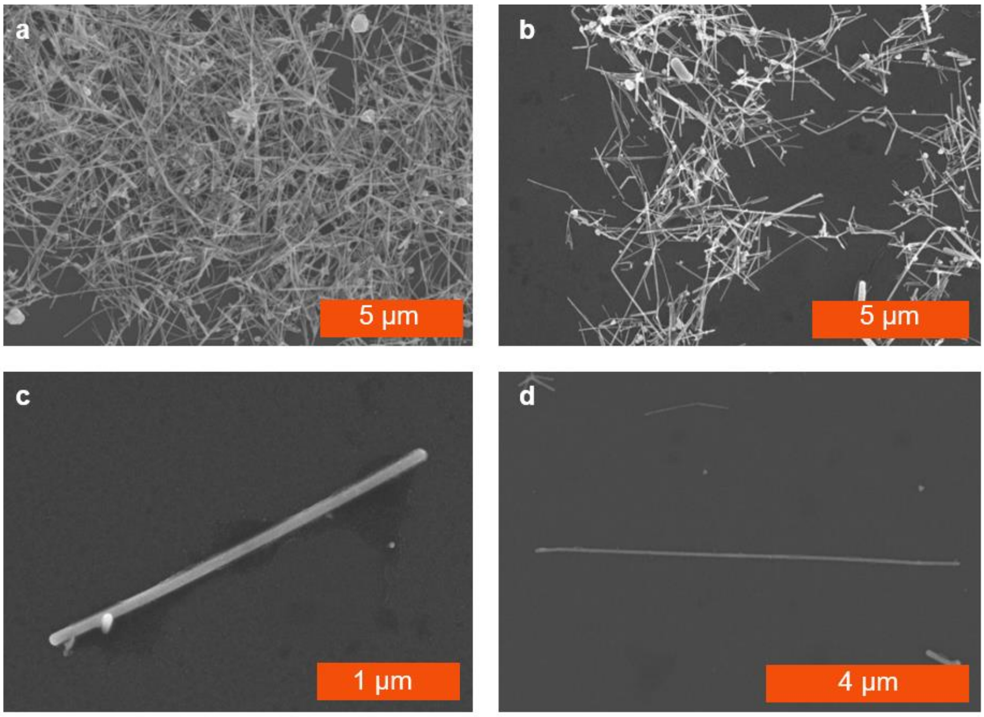

Supplementary Fig. 11. Morphology of Ag nanowires. (a-d) SEM image of Ag nanowires. Scale bar: (a, b) 5 μm; (c) 1 μm; (d) 4 μm.

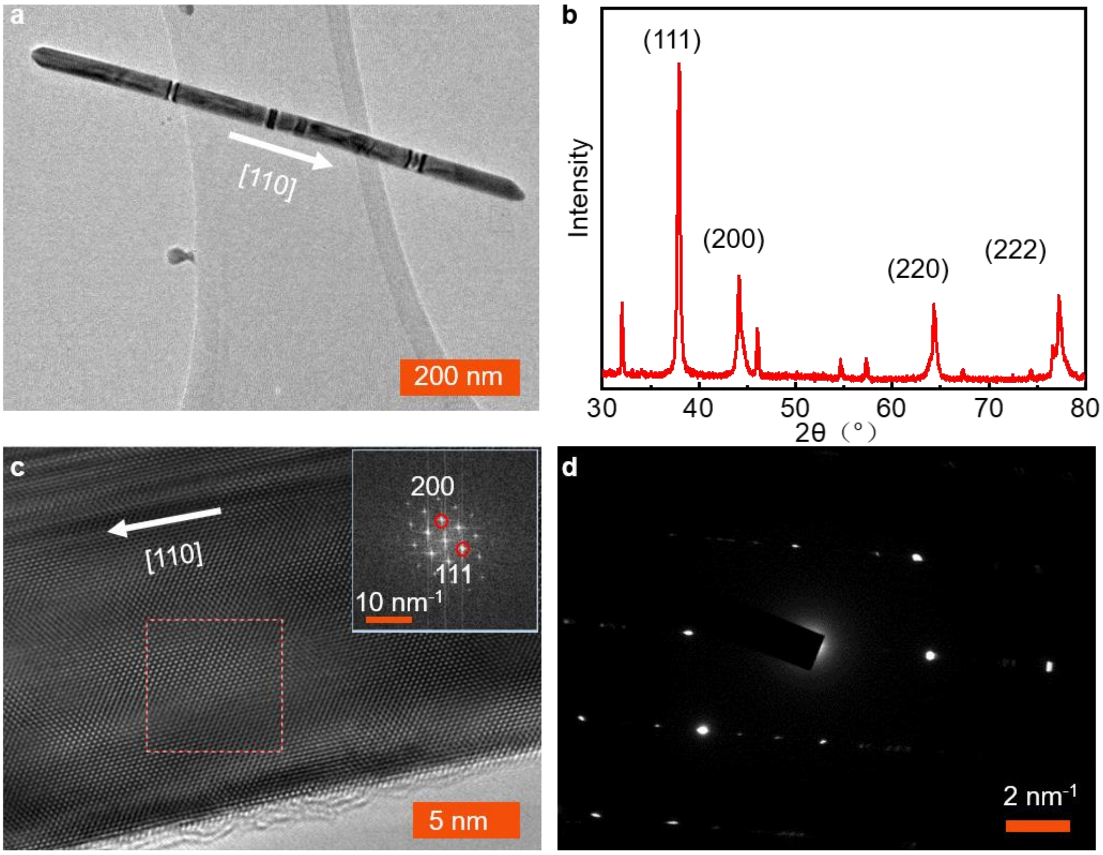

Supplementary Fig. 12. Characterization of Ag nanowires. (a) High-resolution TEM image of a penta-twinned Ag nanowire, which is straight and uniform in diameter with an axial direction of <110>. Scale bar in the inset, 200 nm. (b) XRD pattern of the sample. (c) Fast Fourier transformation (FFT) image of Ag-nanowire lattice fringes. (d) Characteristic SAED patterns of the penta-twinned Ag nanowire.

Supplementary note 3: Penta-twinned Ag nanowires were synthesized by a modified polyol process. Based on TEM characterization, the synthesized nanowires are straight and uniform in diameter, with a growth direction of < 110 >, as shown in Supplementary Fig. 8. The XRD results indicated that the sample is metallic silver (JCPDS file No. 04-0783 from ASTM). The calculated spacing distances were 2.35 and 2.03 Å, which correspond to the <111> and <200> planes of metallic silver (JCPDS file No. 04-0783 from ASTM), respectively. Supplementary Fig. 8d shows the typical selected-area

electron diffraction (SAED) pattern recorded from an individual silver nanowire by aligning the electron beam perpendicular to one of the five side surfaces. This pattern indicated that each silver nanowire was not a single crystal because the diffraction spots could not be assigned to any particular simple pattern associated with face-centered cubic silver.

1.5 Electrochemical reaction process of a AgCl nanowire

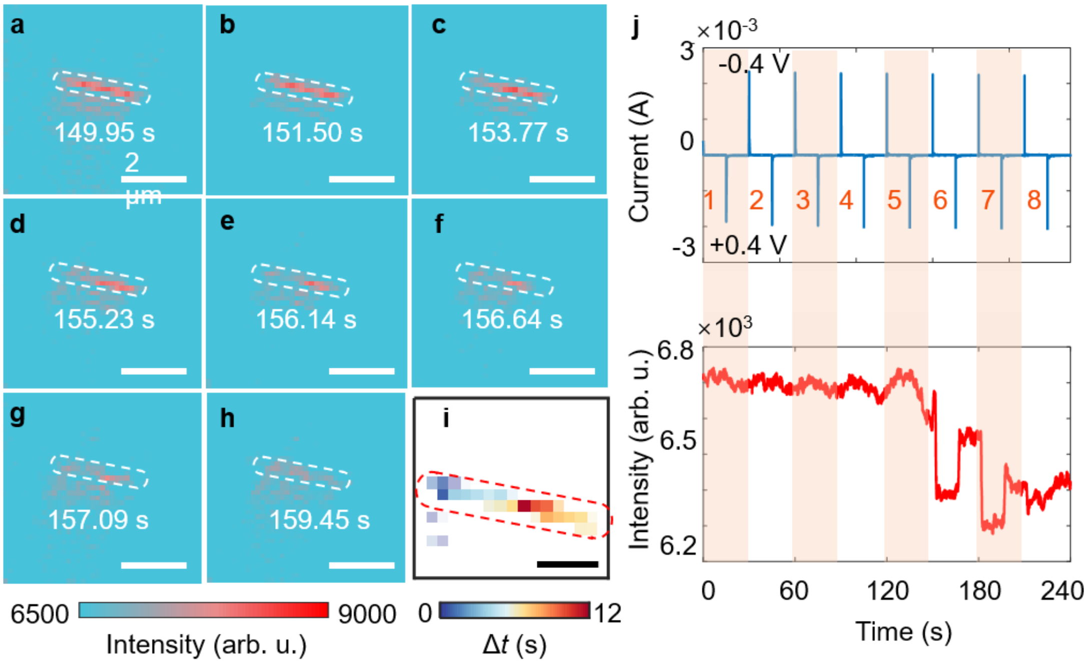

Supplementary Fig. 13. Electrochemical reaction process of a AgCl nanowire. (a-h) Snapshots of a AgCl nanowire captured during the 6th step process. Scale bar: 2 μm. (i) Mapping of the reduction time delay of the 6th step along the nanowire. Scale bar: 400 nm. (j) Current curve and corresponding intensity curve of the whole nanowire during the process.

1.6 Penta-twinned Ag nanowires

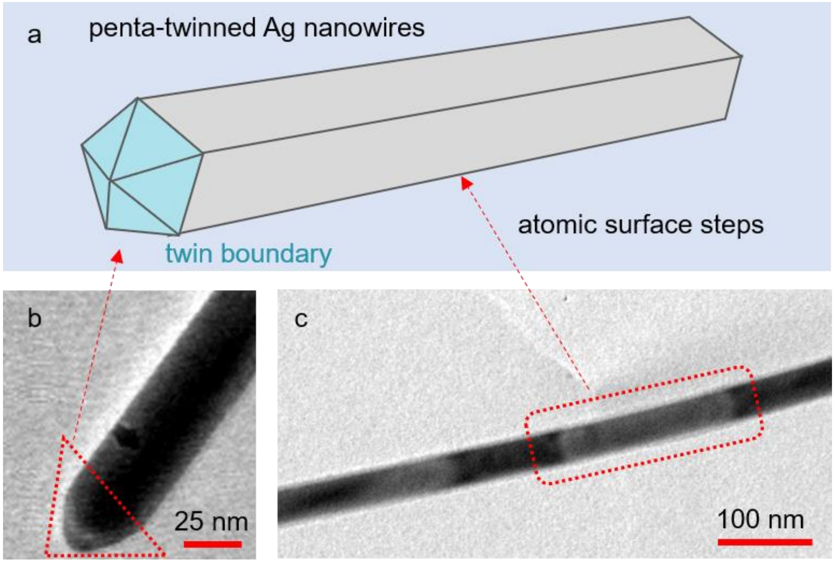

Supplementary Fig. 14. Penta-twinned Ag nanowires. (a) Schematic drawings of a penta-twinned Ag nanowire structure with length direction along the ⟨110⟩ direction. (b) TEM image of the terminus of a Ag nanowire. Scale bar: 25 nm. (c) TEM image of atomic surface steps on a Ag nanowire. Scale bar: 100 nm.

1.7 Electrochemical reaction process of single Ag nanowires of different lengths

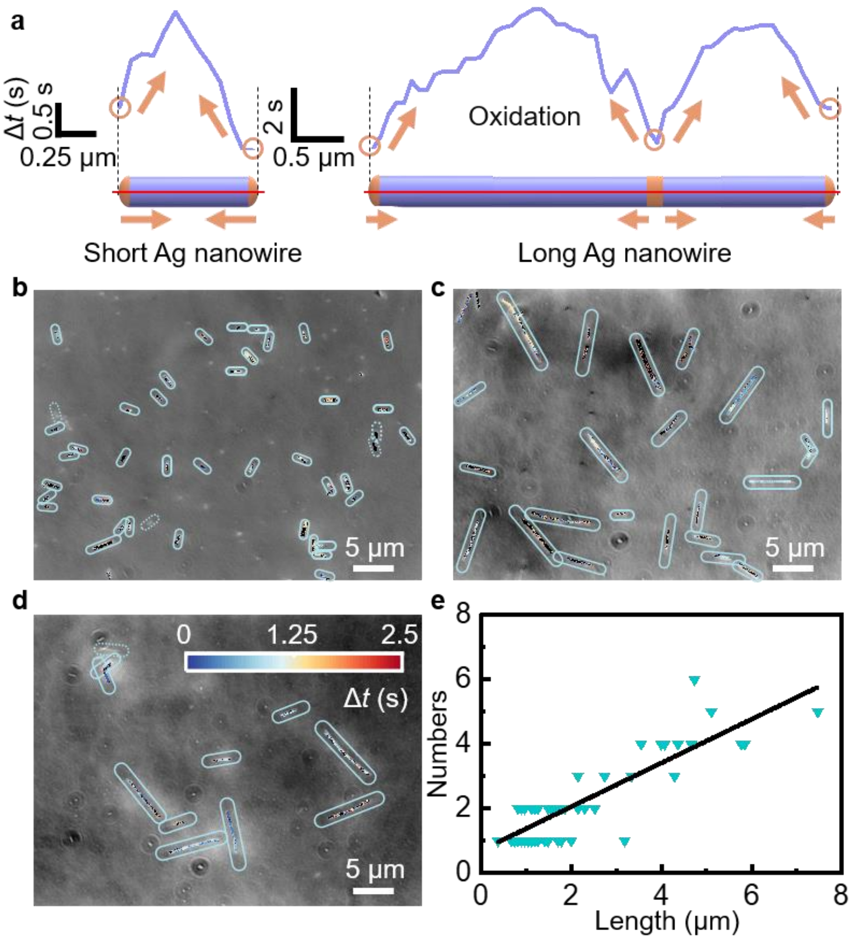

Supplementary Fig. 15. Electrochemical reaction process of single Ag nanowires of different lengths. (a) The oxidation time delay (Δt (s)) was measured at different locations along two nanorods with different lengths (purple line). The schematic diagram illustrates how to determine the number of initial oxidation regions. Merged image of azimuth-modulated plasmonic scattering interferometric images of single short (b) and long (c, d) Ag nanowires along with the corresponding mapping of the original oxidation time delay during the first CV. (e) The relationship between the number of regions of initial oxidation and the length of the Ag nanowires. The black line is the linear fit.

1.8 Characterization of Ag nanowires before/after reaction

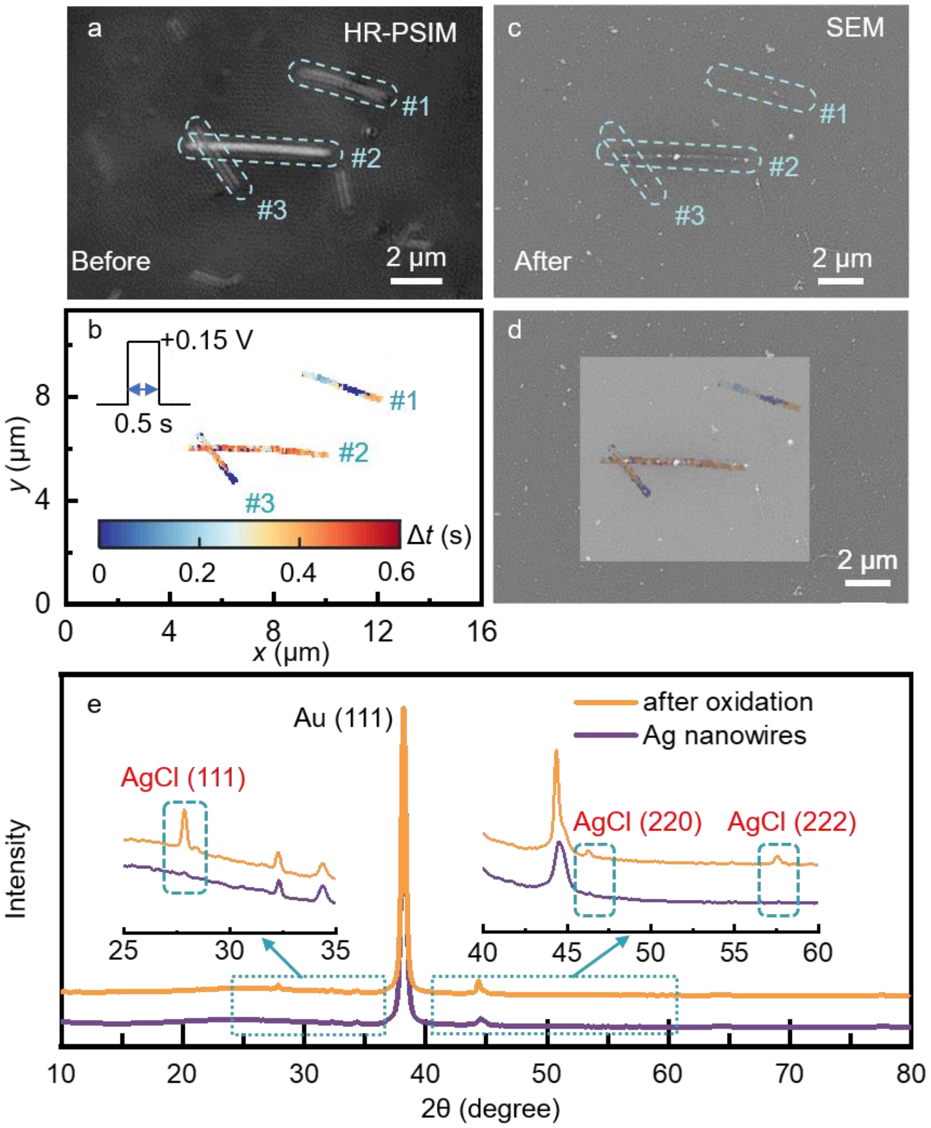

Supplementary Fig. 16. Characterization of Ag nanowires before/after reaction. (a) Azimuth-modulated plasmonic scattering interferometric images of single Ag nanowires. (b) Mapping of the initial oxidation time delay on the nanowires in (a). Inset: Schematic illustration of the voltage changes (+0.15 V for 0.5 s). (c) SEM image of the nanowires at the same view after electro-oxidation. (d) Merged image of (b) and (c). (e) XRD patterns of the Ag nanowires before/after the oxidation reaction. Nanowires were deposited on the surface of the Au chip.

1.9 Tracking the electrochemical dissolution process of a single Ag nanowire

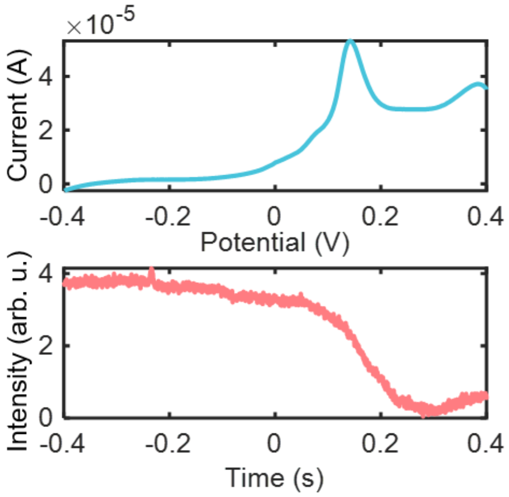

Supplementary Fig. 17. The current signal and corresponding optical signal during the electrochemical dissolution process. The curve of current vs. potential during linear sweep voltammetry (light blue line) and the intensity signal of the nanowire (Fig. 4) from plasmonic scattering interferometric images (magenta line).

1.10 Scheme of the electrochemical chamber

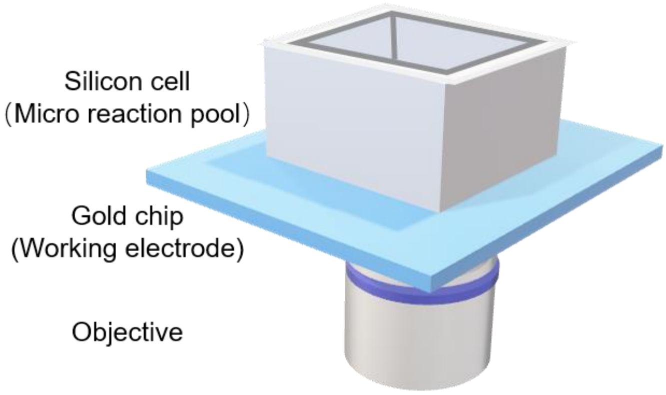

Supplementary Fig. 18. Scheme of the electrochemical chamber.

1.11 Tracking the electrochemical process of single Ag nanowires in KBr solution

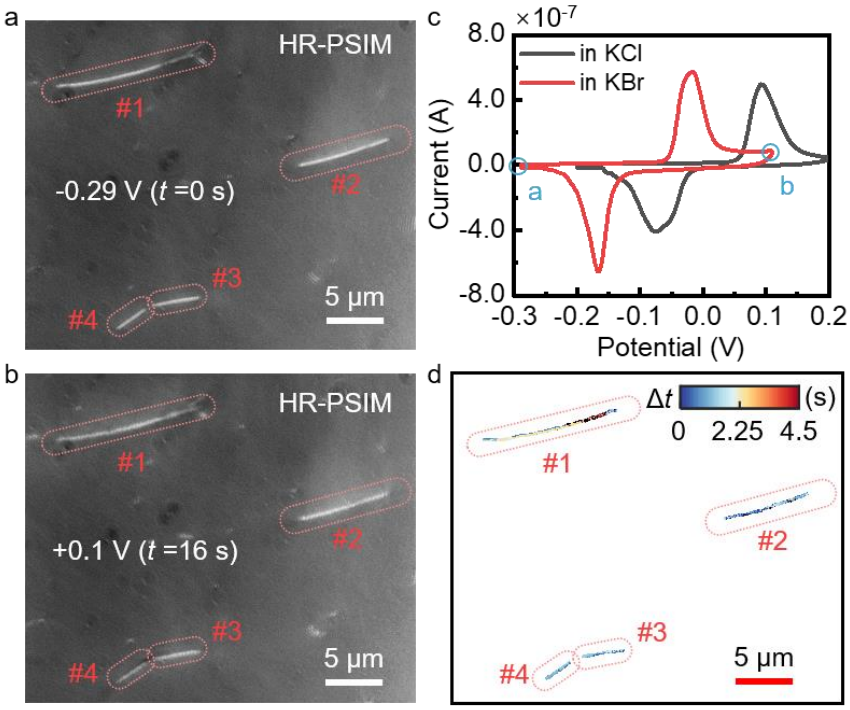

Supplementary Fig. 19. Tracking the electrochemical process of single Ag nanowires in KBr solution. (a) Azimuth-modulated plasmonic scattering interferometric images of single Ag nanowires in KBr solution at a potential of -0.3 V. (b) Corresponding images of the single nanowires in KBr solution at a potential of 0.1 V, where the reaction from Ag to AgBr occurred. (c) Cyclic voltammetry (CV) curves recorded by the potentiostat during the electrochemical reaction on the electrode (black line in KCl solution, red line in KBr solution). (d) Mapping of the initial oxidation time delay on the nanowires during the CV process.

1.12 Data processing

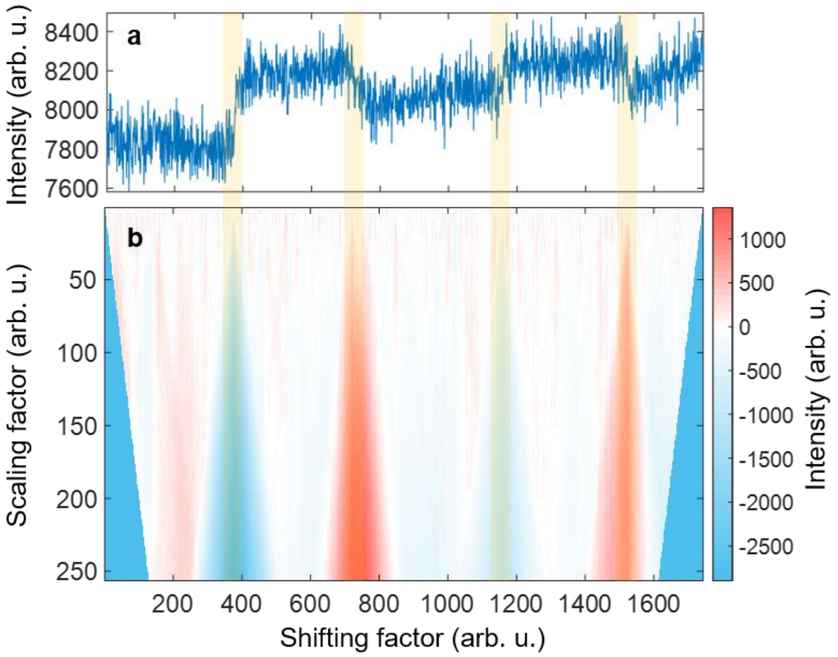

Supplementary Fig. 20. Analysis of the turning point for Ag oxidation/AgCl reduction. (a) A time series of a pixel from the Ag nanowire during cyclic voltammetry (CV) in the range of -0.4 V and +0.4 V. (b) The time-scale space mapped by CWT, indicating that the Ag oxidation/AgCl reduction resulted in a local maxima/minima of the shifting factor.

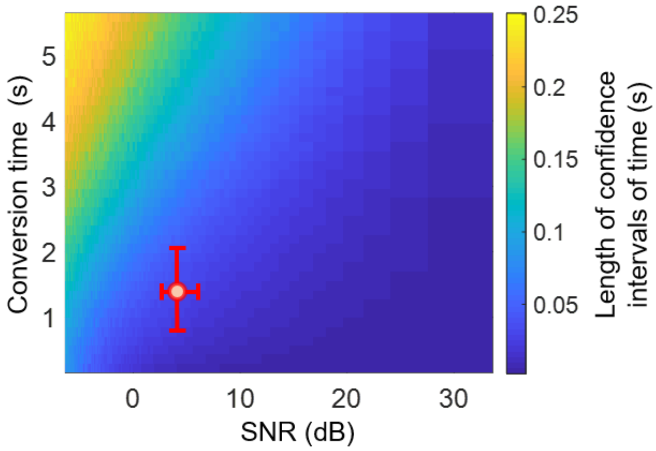

Supplementary Fig. 21. The relationship between the conversion time, SNR and length of confidence intervals of the estimated turning point. The red point shows the conversion time for Ag oxidation/AgCl reduction and SNR under our experimental conditions, indicating that the average length of confidence intervals of the turning point is 11 ms.
