# Supplementary material for: Dynamic imaging of interfacial electrochemistry on single Ag nanowires by azimuth-modulated plasmonic scattering interferometry
Source: Nat Commun. 2023 Jul 13;14:4194. doi: 10.1038/s41467-023-39866-8 (PMC10344930; doi:10.1038/s41467-023-39866-8)
Supplement: Supplementary file 2 — Description of Additional Supplementary Information [file 41467_2023_39866_MOESM2_ESM.docx]

**Description of Additional Supplementary Files**

**Supplementary Movie 1.**

Plasmonic scattering interferometric imaging of the surface reaction dynamics of a single Ag nanowire in KCl solution during the CV process (frame rate: 25 fps).

**Supplementary Movie 2.**

Comparing the behaviors of nanowire A during the two cycles of potential scanning (frame rate: 25 fps).

**Supplementary Movie 3.**

Plasmonic scattering interferometric imaging of the electrochemical dissolution of a single Ag nanowire in NaOH solution (frame rate: 50 fps).
